# Comparative Genomic Analysis and a Novel Set of Missense Mutation of the *Leptospira weilii* Serogroup Mini From the Urine of Asymptomatic Dogs in Thailand

**DOI:** 10.3389/fmicb.2021.731937

**Published:** 2021-10-18

**Authors:** Alongkorn Kurilung, Vincent Perreten, Nuvee Prapasarakul

**Affiliations:** ^1^Department of Veterinary Microbiology, Faculty of Veterinary Science, Chulalongkorn University, Bangkok, Thailand; ^2^Institute of Veterinary Bacteriology, Vetsuisse Faculty, University of Bern, Bern, Switzerland; ^3^Siriraj Metabolomics and Phenomics Center, Faculty of Medicine Siriraj Hospital, Mahidol University, Bangkok, Thailand; ^4^Diagnosis and Monitoring of Animal Pathogens Research Unit, Faculty of Veterinary Science, Chulalongkorn University, Bangkok, Thailand

**Keywords:** comparative genomic analysis, dogs, missense mutation, *Leptospira weilii* serogroup Mini, plasmid

## Abstract

*Leptospira weilii* belongs to the pathogenic *Leptospira* group and is a causal agent of human and animal leptospirosis in many world regions. *L. weilii* can produce varied clinical presentations from asymptomatic through acute to chronic infections and occupy several ecological niches. Nevertheless, the genomic feature and genetic basis behind the host adaptability of *L. weilii* remain elusive due to limited information. Therefore, this study aimed to examine the complete circular genomes of two new *L. weilii* serogroup Mini strains (CUDO6 and CUD13) recovered from the urine of asymptomatic dogs in Thailand and then compared with the 17 genomes available for *L. weilii*. Variant calling analysis (VCA) was also undertaken to gain potential insight into the missense mutations, focusing on the known pathogenesis-related genes. Whole genome sequences revealed that the CUDO6 and CUD13 strains each contained two chromosomes and one plasmid, with average genome size and G+C content of 4.37 Mbp and 40.7%, respectively. Both strains harbored almost all the confirmed pathogenesis-related genes in *Leptospira*. Two novel plasmid sequences, pDO6 and pD13, were identified in the strains CUDO6 and CUD13. Both plasmids contained genes responsible for stress response that may play important roles in bacterial adaptation during persistence in the kidneys. The core-single nucleotide polymorphisms phylogeny demonstrated that both strains had a close genetic relationship. Amongst the 19 *L. weilii* strains analyzed, the pan-genome analysis showed an open pan-genome structure, correlated with their high genetic diversity. VCA identified missense mutations in genes involved in endoflagella, lipopolysaccharide (LPS) structure, mammalian cell entry protein, and hemolytic activities, and may be associated with host-adaptation in the strains. Missense mutations of the endoflagella genes of CUDO6 and CUD13 were associated with loss of motility. These findings extend the knowledge about the pathogenic molecular mechanisms and genomic evolution of this important zoonotic pathogen.

## Introduction

Leptospirosis is a significant zoonotic disease caused by spirochetal bacteria in the genus *Leptospira*. The disease is recognized as a public health concern in many parts of the world, including Thailand (Wuthiekanun et al., 2007; Costa et al., 2015). There are an estimated more than 1.03 million cases and 60,000 deaths from human leptospirosis worldwide per year, and the frequency has tended to increase due to global climate change (Costa et al., 2015). Most mammals can be infected by *Leptospira* with different clinical manifestations ranging from asymptomatic through chronic to acute-life threatening infections (Ko et al., 2009). In addition, asymptomatic animals play a role in disease maintenance and transmission by carrying the pathogen in their kidneys and shedding them *via* their urine, leading to contamination in the environment and infections in humans (Kurilung et al., 2017).

At least 64 different *Leptospira* species have been recognized and divided into three groups based on their phylogeny and virulence status, the latter encompassing the pathogenic, intermediate, and saprophytic groups (Picardeau, 2017; Thibeaux et al., 2018; Casanovas-Massana et al., 2019, 2020; Vincent et al., 2019). Moreover, 24 serogroups with more than 300 serovars have been determined based on the diversity of the lipopolysaccharide (LPS) antigen on the outer cell membrane (Evangelista and Coburn, 2010).

*Leptospira weilii* is a member of the pathogenic *Leptospira* group and has been widely reported to cause disease in humans and animals in Australia, China, Chile, and Thailand (Corney et al., 2008; Mason et al., 2016; Kurilung et al., 2017; Xu et al., 2017). *L. weilii* infection can develop with the classical symptoms of typical leptospirosis in human, such as fever, headaches, and myalgia, similar to that of other pathogenic *Leptospira*, but can also produce pathological changes in the lungs and kidneys in the experimental guinea pig model (Slack et al., 2007; Xu et al., 2017). Nevertheless, two *L. weilii* strains (CUDO6 and CUD13) were recently isolated from the urine of asymptomatic dogs, and to our knowledge, this is the first isolation from a canine source (Kurilung et al., 2017). The characterization of CUDO6 and CUD13 became essential to understand strains that show no infection symptoms in dogs, and serve as a basis for understanding the potential public health impact that such strains may have in the region.

The advent of next-generation sequencing (NGS) over the past decade has accelerated a sudden increase in the number of genome sequences in the database, including for *Leptospira* species. This makes it possible to define genomic information and compare them amongst different groups and/or sources of infection (Fouts et al., 2016). Comparative genome analysis has revealed remarkable knowledge on the genus *Leptospira* in terms of their genes related to pathogenesis and host-adaptation and provided new insights into their genetic variation and evolutionary pathway (Xu et al., 2016). To expand our understanding of genome characteristics along with the evolution process related to host-adaptation, we serotyped and sequenced the *L. weilii* strains CUDO6 and CUD13 using the Oxford Nanopore Technology (ONT) and Illumina NovaSeq platforms to generate complete and accurate whole genome sequences. Multiple bioinformatics tools were then used to characterize their genomic features, and they were then compared with the other available strains. Variant calling analysis (VCA) was also undertaken to investigate missense mutations that affected protein-coding genes related to the pathogenesis of *Leptospira*. This study provides a genetic basis for pathogenic *Leptospira* and improves our understanding of evolution and molecular pathogenesis for further studies.

## Materials and Methods

### Bacterial Strains, Growth Conditions, and DNA Isolation

The genome of 19 *L. weilii* strains that originated from different hosts and geographical locations was used in this study (Table 1), except for *L. weilii* strain ICFT for which the genome was of poor quality and likely to have been species misclassified based on core genome phylogeny and average nucleotide identity (ANI) (Bulach and Adler, 2018; Vincent et al., 2019). The genome of the *L. weilii* strains CUDO6 and CUD13 were obtained during this study, whereas the genome of the 17 other strains was retrieved from the NCBI database for genomic comparison. Strains CUDO6 and CUD13 were recovered from the urine of asymptomatic dogs in Nan province, Thailand, in 2014. Both strains had been isolated in the same period (the year 2014) and in the same area (Sop Khun village, Nan province, Thailand), and were confirmed at the species level by partial *rrs* sequencing and comparative phylogeny. In addition, multilocus sequence typing (MLST) was also examined using seven house-keeping genes (*caiB*, *glumU*, *mreA*, *pfkB*, *pntA*, *sucA*, and *tpiA*) as previously described (Boonsilp et al., 2013). Both strains were assigned as novel sequence type 94 (ST94) belonging to the *L. weilii* cluster. Moreover, the strains shared the single locus variant (*mreA*52) with ST183 (*mreA*49), and ST193 (*mreA*55) from human *L. weilii* isolates in Laos and China (Kurilung et al., 2017).

**TABLE 1:** *Leptospira* strains used for analysis in this study.

Species	Strains	Host	Country	Size (Mbp)	CDSs	G+C (%)	Accession number
*L. weilii*	CUDO6	Dog	Thailand	4.38	3,965	40.8	CP040840.1
*L. weilii*	CUD13	Dog	Thailand	4.36	3,905	40.9	CP040843.1
*L. weilii*	56105	Human	Indonesia	4.03	3,496	40.4	NZ_JQRN00000000.1
*L. weilii*	56145	Human	Malaysia	4.12	3,670	40.8	NZ_JQRO00000000.1
*L. weilii*	56621	Human	China	4.37	3,947	40.6	NZ_JQRP00000000.1
*L. weilii*	56622	Human	China	4.28	3,885	40.7	NZ_JQRQ00000000.1
*L. weilii*	56646	Human	China	4.32	3,905	40.7	NZ_JQRR00000000.1
*L. weilii*	56655	Human	China	4.12	3,731	40.8	NZ_JQSB00000000.1
*L. weilii*	56674	Human	China	4.22	3,861	40.9	NZ_JQQB00000000.1
*L. weilii*	56679	Human	China	4.39	3,975	40.7	NZ_JQQA00000000.1
*L. weilii*	2006001853	Human	Thailand	4.37	3,969	40.9	NZ_AFLV00000000.2
*L. weilii*	2006001855	Human	Thailand	4.27	3,646	40.8	NZ_AFJM00000000.2
*L. weilii*	Ecochallenge	Human	United States	4.03	3,038	40.9	NZ_AHMI00000000.2
*L. weilii*	L231	Human	China	4.26	3,888	40.7	NZ_MSFX00000000.1
*L. weilii*	LNT1194	Human	N/A	4.24	3,870	40.7	NZ_AHQX00000000.1
*L. weilii*	LNT1234	Human	Laos	4.26	3,897	40.8	NZ_AHNC00000000.2
*L. weilii*	LT2116	Human	Australia	4.32	4,981	40.5	NZ_AHOR00000000.2
*L. weilii*	UI13098	Human	Laos	4.55	4,204	40.7	NZ_AHNU00000000.2
*L. weilii*	UT14631	Human	N/A	4.31	3,931	40.7	NZ_AHQY00000000.1
*L. interrogans*	56601	Human	China	4.69	3,683	35	NC_004342.2
*L. kirschneri*	3522 CT	Bat	Indonesia	4.4	3,599	35.9	NZ_AHMN00000000.2
*L. noguchii*	CZ 214	Opossum	Panama	4.71	4,520	35.5	NZ_AKWY00000000.2
*L. alexanderi*	L60	Human	China	4.22	3,571	40.2	NZ_AHMT00000000.2
*L. borgpetersenii*	L550	Human	Australia	3.93	3,288	40.2	NC_008508.1
*L. mayottensis*	200901116	Human	France	4.21	3,544	39.6	NZ_CP024871.1
*L. santarosai*	LT 821	N/A	N/A	3.98	3,673	41.8	NZ_CP006694.1
*L. alstonii*	80-412	Frog	China	4.43	3,868	42.5	NZ_AOHD00000000.2
*L. kmetyi*	Bejo-Iso9	Soil	Malaysia	4.42	4,238	44.8	NZ_AHMP00000000.2
*L. licerasiae*	VAR 010	Human	Peru	4.21	3,974	41.1	NZ_AHOO00000000.2
*L. wolffii*	Khorat-H2	Human	Thailand	4.4	4,061	45.6	NZ_AKWX00000000.2
*L. broomii*	5399	Human	Denmark	4.4	4,205	43	NZ_AHMO00000000.2
*L. inadai*	M34/99	Rat	Brazil	4.55	4,181	44.6	NZ_AHMM00000000.2
*L. fainei*	BUT6	Wild boar	Australia	4.29	4,113	43.5	NZ_AKWZ00000000.2
*L. venezuelensis*	CLM-U50	Human	Venezuela	4.3	4,080	39.2	NZ_NETS00000000.1
*L. biflexa*	Patoc I	Water	Italy	3.95	3,695	38.9	NC_010602.1
*L. yanagawae*	São Paulo	Water	Brazil	4.05	3,771	38.2	NZ_AOGX00000000.2
*L. meyeri*	Went5	N/A	Canada	4.19	3,969	38	NZ_AKXE00000000.1
*L. vanthielii*	Waz Holland	Water	Netherlands	4.23	3,885	38.9	NZ_AOGY00000000.2
*L. wolbachii*	CDC	N/A	United States	4.08	3,912	39.1	NZ_AOGZ00000000.2
*L. terpstrae*	LT 11-33	N/A	China	4.09	3,794	38.2	NZ_AOGW00000000.2
*L. idonii*	201300427	Water	Japan	4.09	3,724	41.1	NZ_RQHW00000000.1

*N/A, no applicable.*

The *L. weilii* CUDO6 and CUD13 strains were kept as a stock culture and stored at −80°C before use. A total of 1 mL of stock culture was inoculated into 10 mL of liquid Ellinghausen–McCullough–Johnson–Harris medium supplemented with 6% (v/v) rabbit serum and incubated at 30°C for 2 weeks. For DNA isolation, the 10-mL cultures were centrifuged at 4,800 rpm for 10 min, and then the genomic DNA was extracted from the cell pellet using the guanidinium thiocyanate-phenol-chloroform extraction method (Pitcher et al., 1989). Subsequently, the obtained DNA was sheared to get DNA fragments ranging from 6 to 20 kbp using Covaris g-TUBE (Covaris, United States). The fragmented DNA was further enriched using Agencourt AMPure XP beads (Beckman Coulter, United States), and measured for quantity and quality (A_260_/A_280_ ratio) using Qubit Fluorometric Quantitation (Thermo Fisher Scientific, United States) and NanoDrop (Thermo Fisher Scientific, United States), respectively. Genomic DNA was stored at −20°C for further library preparation and genome sequencing.

### Motility Assay

For motility testing, 10 μl of mid-log phase cultures of *L. weilii* strains CUDO6 and CUD13 as well as of *Leptospira interrogans* serovar Copenhageni strain M20 (Academic Center of Amsterdam) used as motile control strain were spotted on Difco Leptospira Medium EMJH (Becton, Dickinson and Company) containing 0.5% agar and incubated at 30°C for 5 days. Growth of strains CUDO6, CUD13, and M20 was determined by the measurement of the OD420 after 5 days of incubation in EMJH broth at 30°C.

### Serotyping

The *L. weilii* CUDO6 and CU13 were sent for commercial serotyping at the OIE National Collaborating Centre for Reference and Research on Leptospirosis, Netherlands. For serogroup determination, a total of 43 polyclonal antisera produced from *Leptospira* reference strains were primarily typed using the microscopic agglutination test (MAT) method. Based on the serogroup finding, strains CUDO6 and CUD13 were subsequently typed for serovar level using the MAT technique with a panel of 18 monoclonal antibodies derived from *Leptospira* serogroup Mini coded F13C3, F13C193, F16C28, F16C140, F16C327, F21C2, F22C1, F22C2, F22C6, F28C1, F35C10, F38C13, F38C24, F50C3, F106C1, F106C5, and F106C9.

### Genome Sequencing, Assembly, and Annotation

The DNA library was prepared using the ligation sequencing 1D kit (SQK-LSK108) and barcoded with the Native barcoding kit (EXP-NBD103) (Oxford Nanopore, United Kingdom) as per the manufacturer’s instructions. Whole genome sequencing was performed using a FLO-MIN106 flow cell with the MinION sequencing device (Oxford Nanopore, United Kingdom). Similarly, the genomic DNA was also sent in parallel for short-read DNA sequencing using NovaSeq6000 S2 Reagent Kit (300 cycles) and S2 flow cell on a NovaSeq6000 (2 × 150-bp paired-end) system (Illumina, United States) at Eurofins Genomics GmbH, Germany. Raw fast5 reads from ONT were base called, quality filtered, and demultiplexed using Guppy v2.3.7 (ONT, United Kingdom). Adaptors from the ONT reads were removed using Porechop v0.2.1.^1^ The ONT reads were then assembled using the Canu v1.7 software with the default parameter setting (Koren et al., 2017). Paired-end Illumina reads were paired, trimmed, and quality filtered using the Trimmomatic v0.36 software (Bolger et al., 2014). The quality of the Illumina reads was measured *via* the online FastQC v0.11.8 software.^2^

Subsequently, the Illumina paired reads and draft genome sequence output from the Canu assembler were used as input files for Minimap2 (Li, 2018) and Racon (Vaser et al., 2017) for polishing twice. The third and fourth polishing steps were then performed with Pilon (Walker et al., 2014). For the final inspection and curation, the sequences were manually validated by mapping paired-Illumina reads to the polished sequences with the aid of the Geneious software v10.2.3 (Biomatters, New Zealand). Genome sequences of the strains CUDO6 and CUD13 were primarily annotated with Prokka v1.12 (Seemann, 2014), and the Rapid Annotation using Subsystem Technology (RAST) (Aziz et al., 2008). Metabolic pathways were annotated with KEGG (Kanehisa et al., 2017) and MetaCyc (Caspi et al., 2019), as implemented in the MicroScope platform (Vallenet et al., 2009).

Other online available bioinformatics tools were used to characterize genomic features consisting of phage and genomic island sequences (PHASTER and IslandViewer) (Arndt et al., 2016; Bertelli et al., 2017), CRISPR-Cas system (CRISPRCasFinder) (Couvin et al., 2018), and CRISPR RNA (crRNA) targets (CRISPRTarget) (Biswas et al., 2013). In case of putative virulence factor (VF) identification, all protein sequences of the strains CUDO6 and CUD13 were searched against the VF database (VFDB) (Chen et al., 2016). Moreover, the list of confirmed pathogenesis-related protein sequences of pathogenic *Leptospira* was generated based on the literature (Murray, 2015; Fouts et al., 2016; Gomes-Solecki et al., 2017; Picardeau, 2017). Of these, 34 representative protein sequences that corresponded to the functions of adhesion, dissemination, immune evasion, hemolytic activity, and iron acquisition were retrieved from the UniProt database (UniProt Consortium., 2019) for orthologous protein identification using amino acid sequences. These amino acid sequences were then subjected to orthologous protein similarity searching against all protein sequences of representative *L. weilii* strains using BLASTp with an *e*-value threshold of 1e-06 and a more than 40% identity (Rost, 1999).

The complete nucleotide sequence of *L. weilii* CUDO6 and CUD13 strains was deposited in DDBJ/EMBL/GenBank under the accession numbers CP040840.1 [chromosome I (CI) of CUDO6], CP040841.1 [chromosome II (CII) of CUDO6], CP040842.1 (plasmid of CUDO6), and CP040843.1 (CI of CUD13), CP040844.1 (CII of CUD13), and CP040845.1 (plasmid of CUD13), respectively.

### Average Nucleotide Identity and Phylogenetic Analysis

To confirm and define the species delineation of the CUDO6 and CUD13 strains, the ANI values between each pair of *L. weilii* genomes, and the other public available genome of various *Leptospira* species (Table 1) were calculated using OrthoANI (Lee et al., 2016). Isolates with ANI values of more than 95% in pairwise genome comparisons were considered the same species.

To investigate the phylogenetic relationship of *L. weilii*, the phylogenetic analysis was constructed based on the concatenated sequence of core-single nucleotide polymorphism (core-SNPs) located in the core-genome region of the 19 available *L. weilii* strains (Table 1). Briefly, the 19 *L. weilii* genomes were aligned using the Parsnp program nested in the Harvest package with the default parameter settings (Treangen et al., 2014). The aligned core genome sequence was then filtered for recombination regions using Gubbins (Croucher et al., 2015). The SNP sites were then called and concatenated using the online Snippy v4.4.0 software^3^ and the phylogenetic tree was generated using the maximum likelihood (ML) method with the GTR substitution model and 1,000 bootstrap replicates in the RAxML program (Stamatakis, 2014). The tree was visualized with FigTree.^4^

### Pan-Genome Analysis

The gene repertoire of the 19 *L. weilii* genomes was examined by generating a pan−genome structure using the Anvi’o package following the developer’s recommendation (Delmont and Eren, 2018). Firstly, genome sequences were searched for open reading frames that could encode for proteins using Prodigal v2.6.3 (Hyatt et al., 2010). The proteome sequences of *L. weilii* strains (*n* = 19) were searched for amino acid similarity using the BLASTp algorithm and subsequently clustered as core (shared by all strains), accessory (shared at least one other strain), and unique (presented by only one strain) genes with MCL (Enright et al., 2002). The binary matrix of the presence/absence of gene clusters across all 19 *L. weilii* strains was used to generate fitting pan-genome and core-genome curves using the PanGP software (Zhao et al., 2014). The pan-genome and core-genome curves were derived using a power-law regression model (y = A_*pan*_x^*Bpan*^ + C_*pan*_) and exponential decay model (y = A_*core*_e^*Bcore*^ + C_*core*_), respectively, Tettelin et al. (2005, 2008). The B_*pan*_ or γ parameter of the pan-genome curve indicates whether the pan-genome size of *L. weilii* is open (0 < γ < 1), where the size of the pan-genome increases with additions of new genomes, or closed (γ < 0), where a limited maximum number of genes are found as further genomes are added (Tettelin et al., 2005; Tettelin et al., 2008). Additionally, the unique gene clusters of strains CUDO6 and CUD13 were further inspected and identified using the NCBI and VF databases.

### Variant Calling Analysis

To identify SNPs, VCA was performed using the Snippy v4.4.0 pipeline (see text footnote 3). Briefly, the filtered Illumina reads of *L. weilii* strains CUDO6 and CUD13 were mapped against the reference genome of *L. weilii* strain 2006001853 (human isolate) using BWA-MEM v0.7.12 (Li and Durbin, 2009). The SNPs were subsequently identified using Freebayes v1.1 with the default parameter setting.^5^ The missense variants that potentially affected the protein sequences were annotated using SnpEff v4.3 (Cingolani et al., 2012) and the EggNOG database (Huerta-Cepas et al., 2017).

## Results and Discussion

### Serotyping of *Leptospira weilii* CUDO6 and CUD13 Strains

A panel of polyclonal antibodies representing all pathogenic and saprophytic *Leptospira* serogroups were primarily typed for serogroup classification of *L. weilii* strains CUDO6 and CUD13. Both strains were reacted to polyclonal antibodies producing from *Leptospira* serogroups Hebdomadis, Mini, and Sejroe. Nevertheless, they showed the highest MAT titer against *Leptospira* serogroup Mini (titer > 1:1,280) than that of other serogroups (Supplementary Table 1).

For serovar identification, the whole set of monoclonal antibodies designing to differentiate serovars of serogroup Mini was subsequently typed in both strains. The results indicated that *L. weilii* CUDO6 and CUD13 strains reached the highest MAT titer against monoclonal antibody F106C1 (titer = 1:640) with undesignated serovar identification (Supplementary Figure 1). Moreover, both strains shared a similar agglutination pattern to all monoclonal antibodies typing, suggesting that CUDO6 and CUD13 strains might be the same serovar and were likely to recognize as a novel serovar in dogs. They also had closely antigenic relatedness to undesignated serovar of *Leptospira mayottensis* serogroup Mini strain 200901116, supporting by closely agglutination pattern of all monoclonal antibodies typing (Supplementary Figure 1).

### General Genomic Feature of the *Leptospira weilii* CUDO6 and CUD13 Strains

Analysis of the genomic features of *L. weilii* strains CUDO6 and CUD13, recovered from the urine of asymptomatic dogs, showed that the strains were comprised of three circular replicons with a similar G+C content of 40.9%, which accounted for a total size of 4.38 and 4.36 Mbp for CUDO6 and CUD13, respectively. Of these, the two large replicons of strains CUDO6 (3.96 and 0.32 Mbp) and CUD13 (3.97 and 0.28 Mbp) were referred to as CI and CII, respectively. The small circular replicons contained 89.23 kbp for strain CUDO6 and 95.48 kbp for strain CUD13 with an average G+C content of 37.28%. These small circular replicons confirmed the previous report of plasmids in *L. weilii* (Wang et al., 2015), and represent, to the best of our knowledge, the first release of a complete circular plasmid sequence in *L. weilii*. The two plasmids were designated pDO6 and pD13 for the strains CUDO6 and CUD13, respectively.

**FIGURE 1:**
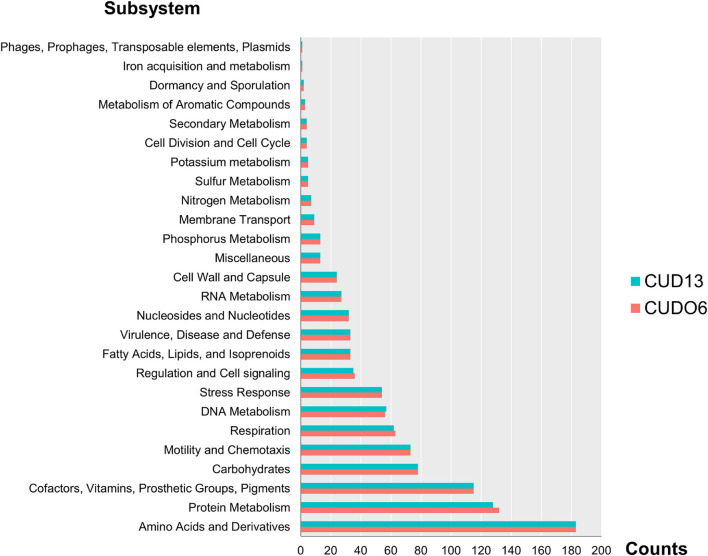
Number of RAST subsystems annotated in the *L. weilii* serogroup Mini strains CUDO6 (pink bar) and CUD13 (blue bar). The CDS of strains CUDO6 and CUD13 were classified into 26 functional subsystems, and arranged according to the number of CDS in each category. Of these, amino acid and derivatives, protein metabolism, and cofactor had the largest number of identified CDS in both *L. weilii* genomes.

The *L. weilii* CUDO6 genome encoded for 3,965 protein-coding sequences (CDSs), whilst the *L. weilii* CUD13 genome yielded 3,905 CDSs. Of the predicted proteins, 1,006 CDSs and 1,001 CDSs, respectively, were function-categorized into 26 subsystems by RAST annotation. Both strains had a broadly similar number of proteins related to the subsystem of amino acids and derivatives, protein metabolism, cofactors, vitamins, prosthetic groups, pigments, carbohydrates, and motility and chemotaxis (Figure 1 and Supplementary Table 2).

**FIGURE 2:**
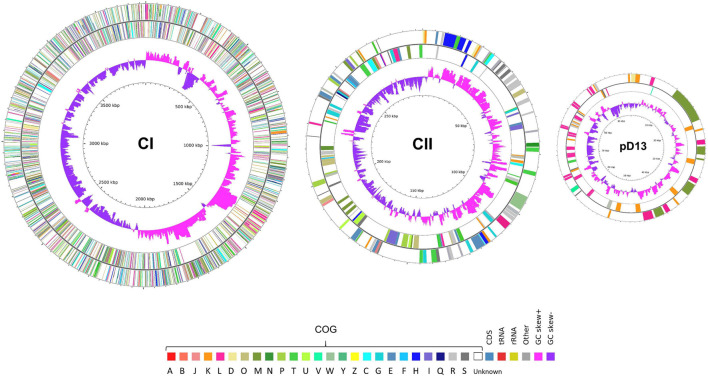
Circular maps of three replicons of *L. weilii* serogroup Mini strain CUD13. The outermost and second outermost rings represent genes on the sense and antisense strands colored according to COG categories annotation. The COG categories are denoted as follows: A (RNA processing and modification), B (chromatin structure and dynamics), J (translation, ribosomal structure and biogenesis), K (transcription), L (replication, recombination, and repair), D (cell cycle control, cell division, and chromosome partitioning), O (post-translational modification, protein turnover, and chaperones), M (cell wall/membrane/envelope biogenesis), N (cell motility), P (inorganic ion transport and metabolism), T (signal transduction mechanisms), U (intracellular trafficking, secretion, and vesicular transport), V (defense mechanisms), W (extracellular structures), Y (nuclear structure), Z (cytoskeleton), C (energy production and conversion), G (carbohydrate transport and metabolism), E (amino acid transport and metabolism), F (nucleotide transport and metabolism), H (coenzyme transport and metabolism), I (lipid transport and metabolism), Q (secondary metabolites biosynthesis, transport, and catabolism), R (general function prediction only), and S (function unknown). The second inner ring indicates the GC skew; positive skew is shown in pink-purple, and negative skew is shown in purple. The genome map was created and visualized using CGView (Stothard and Wishart, 2005).

The putative replication origin of CI and CII in both strains was predicted from the GC skews (Supplementary Figure 2). Similar to the other *Leptospira* species, the replication origin of CI was adjacent to the *dnaA*, *dnaN*, *recF*, and *gyrAB* genes (Ren et al., 2003; Nascimento et al., 2004b; Bulach et al., 2006; Picardeau et al., 2008), while replication origin of CII and the plasmid were adjacent to the partitioning system genes (*parAB*) and downstream of the replication gene (*repB*) (Bulach et al., 2006). Five rRNA genes, comprised of one *rrf*, two *rrl*, and two *rrs* genes, were identified in CI of CUDO6 and CUD13. Moreover, they had a similar number of tRNA genes (*n* = 37), encoding for all 20 amino acids. The chromosomal mapping based on the COG category of *L. weilii* strain CUD13 is illustrated in Figure 2, and the general genome characteristics of *L. weilii* CUDO6 and CUD13 are presented in Table 1.

From the KEGG and MetaCyc metabolic pathway analysis, genes encoding for the enzymes for biosynthesis of all 20 amino acids and glycolysis were present in both CUDO6 and CUD13 strains. In addition, genes responsible for long-chain fatty acid β-oxidation and glycerol degradation were also found, including acetyl-CoA acyltransferase, 2,3,4-saturated fatty acyl-CoA synthetase, enoyl-CoA hydratase, acetyl-CoA *C*-acyltransferase, glycerol kinase, and glycerol 3-phosphate oxidoreductase. Furthermore, genes involved in the tricarboxylic acid cycle, encompassing genes encoding for pyruvate dehydrogenase, malate dehydrogenase, and citrate synthase, were identified. The gene for pyruvate dehydrogenase could synthesize the intermediate product of acetyl-CoA, whilst malate dehydrogenase and citrate synthase could catalyze the reduction of malate and oxaloacetate to oxaloacetate and citrate, respectively (Supplementary Figure 3). These findings supported that both *L. weilii* strains could utilize fatty acid and glycerol as energy sources similar to those of all *Leptospira* species (Fouts et al., 2016).

The genome structure comparison between CUDO6 and CUD13 showed that both strains extensively shared syntenic region in CI, although insertion sequence (IS) mediated chromosomal rearrangements were observed in strain CUDO6. The major rearrangement event in CUDO6 CI occurred at around 1.40 Mbp from the replication origin. This region had two identical copies of IS*Lbp6* (*fhg67_rs07085* and *fhg67_rs14785*) facing in opposite orientation at both sides of the inversion breakpoints, resulting in inverted sequence of a fragment of approximately 1.75 Mbp (Supplementary Figure 4). In contrast, the CII had a nearly colinear structure, except for an ∼39 kbp insertion region in CUDO6, which likely resulted from phage-mediated horizontal gene transfer (Supplementary Figures 2, 4). Plasmid comparison showed highly sequence similarity, except for an 8 kbp insertion region in pD13, which contained genes encoding for hypothetical proteins (Supplementary Figure 4).

Although CUDO6 and CU13 belong to the same ST (ST94), genomic organization of both strains were different. This finding reflects the plasticity of genomic structure in *Leptospira* species (Jorge et al., 2018).

### Phage, CRISPR-Cas System, and Putative Virulence Factor

A total of six and five putative prophage sequences were predicted in the CUDO6 and CUD13 genomes, respectively (Supplementary Table 3). The putative prophage sequences had a size range from 7.4 to 21.8 kbp.

One intact prophage was found in CI of both CUDO6 and CUD13, flanking by *attL* and *attR* with an estimated size 16.05 and 10.27 kbp in length, respectively, and a G+C content of around 41%, which was higher than that of the three circular replicons. They harbored a similar number of CDSs (*n* = 18), which were conserved between the two *L. weilii* strains. The prophage region was predicted to have an attachment site (*attL* and *attR*), putative tail fiber protein, lysis protein, phage-like proteins (PLPs), transposase, and hypothetical proteins that were homolog in sequence to many known prophage sequences, such as *Arthrobacter* phage vB_ArtM-ArV1, *Burkholderia* phage KS9, Stx2-converting phage 1717, and *Listeria* phage LP-114. Intriguingly, one cryptic incomplete prophage was found within the serovar determinant region (*rfb* locus) of the CUDO6 and CUD13 genomes, spanning around 10.5 kbp and encoding for 10 CDSs. Of these, four CDSs shared a 30–63% amino acid identity with the rhamnose biosynthesis enzymes of *Burkholderia vietnamiensis* phage G4, three CDSs matched (33–35% amino acid identity) to the glycosyltransferase genes of *Staphylococcus aureus* phage N315, and one CDS matched (27% amino acid identity) to the DegT family aminotransferase of *Methylobacterium extorquens* phage PA1. All CDSs related to O-antigen biosynthesis loci found on the prophage may be responsible for phage infection and/or the serogroup/serovar diversity in *Leptospira* species. Previously, O-antigen was demonstrated as an important receptor for leptophage infection (Schiettekatte et al., 2018). Additionally, phage infection has been also reported to play an essential role in the emergence of novel serotypes in *Salmonella* spp., *Shigella flexneri*, and *Vibrio cholera* (Wright, 1971; Allison and Verma, 2000; Faruque et al., 2003). Thus, this cryptic prophage may exploit O-antigen as a receptor and also mediate O-antigen variation and/or serovar conversion in *Leptospir*a by altered sugar composition and/or sugar ramification on the LPS structure (Allison and Verma, 2000). The effect of prophage on the *Leptospira*, especially to O-antigen alteration and/or serotype diversity, remains however to be evaluated.

In the CUDO6 genome, PHASTER and IslandViewer also identified a 39.23 kbp prophage-associated genomic island sequence in CII that encoded for 62 CDSs. This result correlated with the larger size of CII in strain CUDO6 comparing with that in strain CUD13. The ∼39 kbp-prophage-associated genomic island carried attachment site (*attL* and *attR*), PLPs involved with the phage baseplate protein (*fhg67_rs19690*), phage tail protein (*fhg67_rs19725*), phage virion morphogenesis protein (*fhg67_rs19755*), phage head morphogenesis protein (*fhg67_rs19790*), phage terminase protein (*fhg67_rs19800*), transcriptional regulation (*iclR*), integrase protein (*fhg67_rs19900*), and host-nuclease inhibitor protein (*fhg67_rs19925*).

The PLP that encoded for the host-nuclease inhibitor protein matched (27% amino acid identity) to the *Haemophilus* phage SuMu. The host-nuclease inhibitor protein is responsible for inhibiting the host RecBCD proteins, one of the host defense mechanisms for bacteriophage infection. This putative prophage may use this protein to interfere the helicase and nuclease activities of RecBCD, enabling the phage to escape DNA degradation during phage replication (Court et al., 2007). In addition, plasmids pDO6 and pD13 were found to both have a similar intact prophage sequence with a total length of 21.8 kbp that encoded for 37 CDSs. Most of these CDSs were involved with the transcriptional regulatory system and post-segregational killing system. These proteins may assist both *L. weilii* strains adapt their growth and facilitate colonization in various milieus (Engelberg-Kulka and Glaser, 1999).

**FIGURE 3:**
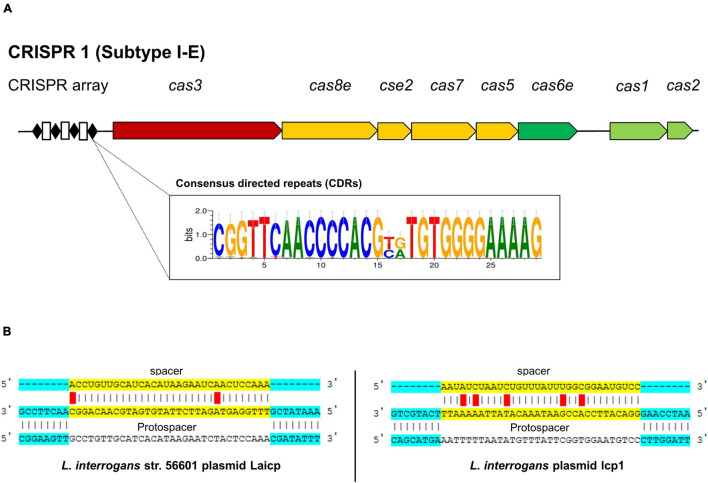
CRISPR-Cas gene organization in the *L. weilii* serogroup Mini strains CUDO6 and CUD13. Both *L. weilii* strains contained CRISPR1 subtype I-E with an uncoupled CRISPR array region located upstream of the locus **(A)**. The CRISPR arrays harbor both of the consensus directed repeats (black diamond) and spacers (white rectangle). Strains CUDO6 and CUD13 carried two different consensus directed repeats, as shown in the sequence logo box **(A)**. DNA sequences in the spacers are transcribed into CRISPR RNA (crRNA) and targeted to the protospacer of invaded foreign DNA. The spacers of strains CUDO6 and CUD13 have the highest matching score to the protospacer of *Leptospira* plasmid Laicp and lcp1 **(B)**.

A cluster of regulatory interspaced short palindromic repeats and CRISPR-associated genes or CRISPR-Cas systems are an adaptive immunity of bacteria that confer resistance to foreign exogenous nucleic acid invasion, such as bacteriophages and plasmids (Makarova et al., 2015). These systems are found in around 40% of bacterial populations, including *Leptospira* species (Fouts et al., 2016; Makarova et al., 2020). This study used the criteria that broadly divided the CRISPR-Cas system into two classes (class 1 and class 2), with further subdivision into 33 distinct subtypes based upon the variety of the *cas* gene combination, sequence similarity, and phylogenetic analysis (Makarova et al., 2020). Both *L. weilii* strains CUDO6 and CUD13 harbored only one CRISPR loci, which was subsequently identified to CRISPR-Cas class 1 (CRISPR1) subtype I-E (Figure 3A). This CRISPR comprised eight *cas* genes (*cas1*, *cas2*, *cas3*, *cas5*, *cas6e*, *cas7*, *cas8e*, and *cse2*) encoded for adaptation, expression, and interference modules for foreign DNA invasion (Al-Attar et al., 2011). The CRISPR arrays of both *L. weilii* strains were uncoupled with the *cas* genes cluster and localized upstream of the CRISPR loci. The CRISPR arrays consisted of two similar consensus directed repeats (CDRs) but with a different number of spacers (32 and 31 for CUDO6 and CUD13, respectively). Both *L. weilii* strains shared 31 identical spacers in their CRISPR arrays (Supplementary Table 4), with one unique spacer found in strain CUDO6 that was identified only as a hypothetical protein.

The shared spacers of the CRISPR arrays of CUDO6 and CUD13 may reflect the same immunity background and evolutionary pathway of the two strains by sharing the same environmental challenges before dissemination to the new host. For a more detailed analysis, the spacer sequences of both *L. weilii* were screened for a putative protospacer that matched to the phage/prophage and plasmid sequence using the CRISPRTarget web tool (Biswas et al., 2013). Among the 31 identical spacers, four protospacers were targeted to *Leptospira* phages and plasmids, including *Leptospira* phage LalZ_80412, *L. interrogans* plasmid Laicp, *L. interrogans* plasmid lcp1, and *L. mayottensis* plasmid p_Lmay_MDI272 (Figure 3B). The result suggested that these phages and plasmids may successfully enter *L. weilii*.

**FIGURE 4:**
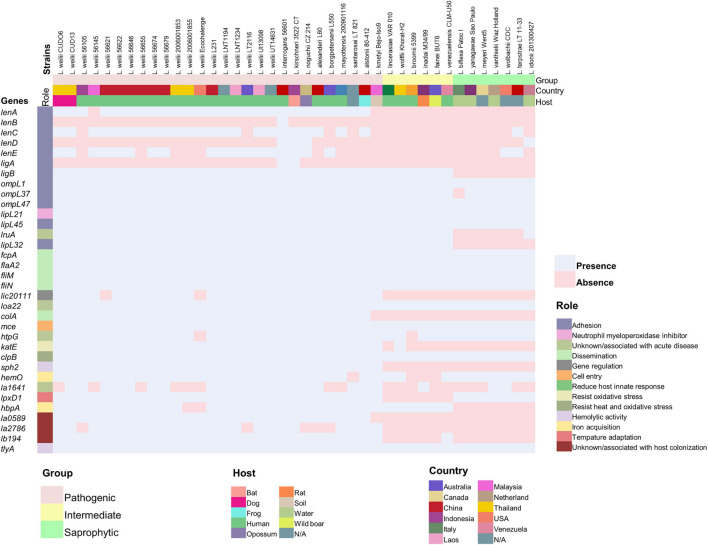
Distribution of the 34 representative confirmed virulence genes across 41 strains of pathogenic, intermediate, and saprophytic *Leptospira*. The representative confirmed virulence genes in each strain were determined using the deduced amino acid sequence obtained from the UniProt database and BLASTp algorithm. Presence and absence of genes are represented by colored pale pink and pale blue boxes, respectively. The distinct *Leptospira* group, source of isolation (country and host), and role of the confirmed virulence genes are represented by different colors. The *L. weilii* serogroup Mini strain CUD13 contained all of the representative confirmed virulence genes, while strain CUDO6 contains all except the gene corresponding to acute infection (*la1641*).

According to the BLAST search of the VFDB, a total of 518 and 519 putative VFs were found in strains CUDO6 and CUD13, respectively (Supplementary Tables 5, 6). Most of the VFs were broadly categorized into adherence, motility, chemotaxis, and secretion systems. To gain insight into the main pathogenesis-related proteins in *Leptospira*, a total of 34 representatives experimentally confirmed VFs were recruited from the literature (Murray, 2015; Fouts et al., 2016; Picardeau, 2017), and protein sequence homology searching with the BLASTp algorithm revealed that 23 proteins (LigB, OmpL1, OmpL37, OmpL47, LipL21, LipL45, LruA, LipL32, FcpA, FlaA2, FliM, FliN, Loa22, ColA, Mce, KatE, ClpB, Sph2, HemO, LpxD, LA0589, LB194, and TlyA) were similarly distributed amongst all analyzed *L. weilii* strains (*n* = 19), indicating that those VFs were commonly shared in all *L. weilii* (Figure 4 and Supplementary Table 7). Strains CUDO6 and CUD13 contained almost all of the confirmed VFs. Nevertheless, proteins LenB, LenD, and LigA for adherence function were absent in both strains, like those reported for other *L. weilii* strains. Protein LA1641, which is associated with LPS structure was also absent in CUDO6 (Murray et al., 2010).

Although strains CUDO6 and CUD13 had genes with confirmed roles as VFs, they were isolated from the urine of asymptomatic dogs. Thus, a balance of gene contents and gene expressions may provide a better explanation than the simple gene presence or absence for bacterial phenotypes. The process of host-adaptation is hypothesized to include both events. Therefore, virulence genes present in the *L. weilii* genome may be down-regulated during host-adaptation, thereby facilitating bacterial fitness to establish persistence in dog kidneys.

### *rfb* Locus, Sialic Biosynthesis, and Lipid a Biosynthesis Encoding Genes

According to the serotyping, both the CUDO6 and CUD13 *L. weilii* strains were identified as belonging to the serogroup Mini (Supplementary Table 1). Subsequently, serovar typing was homologous to the undesignated serovar, which was serologically closely related to the undesignated serovar of *L. mayottensis* serogroup Mini strain 200901116 (Bourhy et al., 2014). We next investigated serovar determinant region (*rfb* locus) of the strains CUDO6 and CUD13, and compared these genetic contents with the same (serogroup Mini) and different serogroups (serogroups Hardjo and Icterohaemorrhagiae) to find the link between serotyping and genetic information.

Strains CUDO6 and CUD13 harbored a serovar determinant region (*rfb* locus) spanning around 93 kbp in the genome and encoding for 87 CDSs (Supplementary Figure 5). Consistent with previous reports, their *rfb* loci were embedded between the conserved MarR transcriptional regulator protein and the DASS sodium-coupled anion symporter, localized upstream and downstream of the *rfb* loci, respectively (Fouts et al., 2016). The complete genes of the dTDT-rhamnose biosynthesis (*rfbABCD*) were found in their *rfb* loci, similar to that in other pathogenic *Leptospira* species (Fouts et al., 2016). Therefore, this typical gene cluster may be necessary for pathogenesis by altering the complexity of the LPS structure (Patra et al., 2015).

The Wzx/Wzy-dependent pathway associated with the O-antigen assembly and export system were subsequently found amongst the loci of both strains, including the flippase gene *wzx* and the O-antigen polymerase gene *wzy*. The sialic biosynthesis gene for *N*-acetylneuraminic (sialic) synthase (*neuB2*) was also identified within the loci. This gene is involved in producing sialic acid and its derivatives (legionamic and pseudaminic acids), which participate in the post-translational modification of surface proteins, and are involved in bacterial colonization, immune evasion, and biofilm formation (Fouts et al., 2016). Therefore, the sialic biosynthesis genes may facilitate CUDO6 and CUD13 to persist as asymptomatic infection agents in dogs. Although the lipid A biosynthesis encoding genes were not situated within this region, a complete set of 13 genes (*lpxA*, *lpxB1*, *lpxB2*, *lpxC*, *lpxD1*, *lpxD2*, *lpxK*, *kdsA*, *kdsB1*, *kdsB2*, *kdtA*, *lnt*, and *htrB*) involved in the pathway were identified elsewhere in both *L. weilii* genomes (BLASTp analysis).

In comparison, both *rfb* loci of the CUDO6 and CUD13 strains showed a highly conserved sequence homology in gene compositions and organization (sharing of 87 paired orthologous proteins). Moreover, a total of 73 orthologous genes were conserved with the closely related (by serotyping) strain of *L. mayottensis* serogroup Mini strain 200901166 (Supplementary Table 8), and had difference of genetic contents in *rfb* locus with distinct serogroups [serogroups Hardjo and Icterohaemorrhagiae (Supplementary Figure 5)]. This finding reflected that the gene contents in the *rfb* loci were shared among some antigens/epitopes in the same serogroup, supporting the previous serotypic observation that found serologically indistinguishable serotypes would share highly identical gene contents amongst their *rfb* loci even though they came from different *Leptospira* species (de la Pena-Moctezuma et al., 1999).

Nevertheless, there were examples of unique genes in the *rfb* loci of CUDO6 and CUD13 that were not found in *L. mayottensis* strain 200901166, although most of these unique genes encoded for hypothetical proteins. Changes in gene contents in this region are thought to be related to serovar diversity and may lead to variation in the serological characteristics (de la Pena-Moctezuma et al., 1999). In addition, four genes from the DegT family of aminotransferases were found in the *rfb* loci of both CUDO6 and CUD13. This protein may allow the biosynthesis of the O-antigen side chains and contribute to the unique LPS structure of strains CUDO6 and CUD13 (Nascimento et al., 2004a).

### Characteristics of the Extrachromosomal Chromosome (Plasmid) of *Leptospira weilii*

The pDO6 and pD13 replicons contained 100 and 108 protein-coding genes (CDSs) with an overall G+C content of 37.40 and 37.20%, respectively. Both replicons had a lower G+C content than the circular chromosomes, which is congruent with the low G+C content of the confirmed plasmid Laicp in *L. interrogans* (34.64%) (Huang et al., 2014) and plasmid p74 in *Leptospira biflexa* (37.47%) (Picardeau et al., 2008). Additionally, the two extrachromosomal DNA replicons met the criteria of being independently replicating plasmids, since their genome harbored CDSs related to the replication region (predicted by GC skew), partition system (*parA* and *parB*), and toxin/antitoxin system (TAS) (*mazE* and *mazF*), which concurs with the previous report of plasmid characterization in *L. interrogans* (Zhu et al., 2014). Moreover, a transcriptional regulator, transposase, and recombinase were identified in both plasmids. Notably, neither resistance genes nor specific virulence genes were detected within pDO6 and pD13, and they showed no similarity to the other *L. weilii* genomes in the database. However, some regions shared similarities with plasmids and chromosomes in other *Leptospira* species (i.e., *L. interrogans* and *L. mayottensis*), suggesting that the *Leptospira* plasmids might share some epitopes and could integrate some of their DNA into the chromosome.

When pDO6 and pD13 were compared with the plasmid Laicp and p74, no orthologous gene cluster was shared amongst the four analyzed plasmids. However, 15 orthologous gene clusters that mainly encoded for DNA replication protein, DNA-binding domain, MazEF toxin-antitoxin module, and transposase were common in the pathogenic *Leptospira* plasmid (pDO6, pD13, and Laicp) (Supplementary Table 9), suggesting that plasmids of saprophytic and pathogenic *Leptospira* might use a different mechanism to be maintained in the respective host cell. A total of 65 orthologous gene clusters were shared amongst plasmids pDO6 and pD13, including genes encoding for chromosome segregation protein, transcriptional regulator, and HigAB toxin-antitoxin module. Plasmid pD13 had six unique genes, which all encoded for hypothetical proteins. Within both *L. weilii* plasmids, the TASs may play a functional role in the stress response during persistence in nutritionally limited terrestrial environments or in the kidneys, rather than a role in maintaining plasmid stabilization during cell division (Christensen et al., 2003). Therefore, plasmids that carry beneficial genes for survival may promote *L. weilii* adaptability to a specific habitat.

### Phylogenetic Analysis and Average Nucleotide Identity

The ANI values from the pairwise genome comparisons within intragenic *L. weilii* strains were all above 95%, whereas the DNA–DNA relatedness comparisons with any two interspecies strains varied substantially from 64.69 to 94.41%. The ANI values supported the taxonomic status of strains CUDO6 and CUD13 as *L. weilii* species, as presented by the ANI values above 95% of the threshold for species delineation. Moreover, strains CUDO6 and CUD13 shared the highest ANI values (99.86%) (Supplementary Table 10).

**FIGURE 5:**
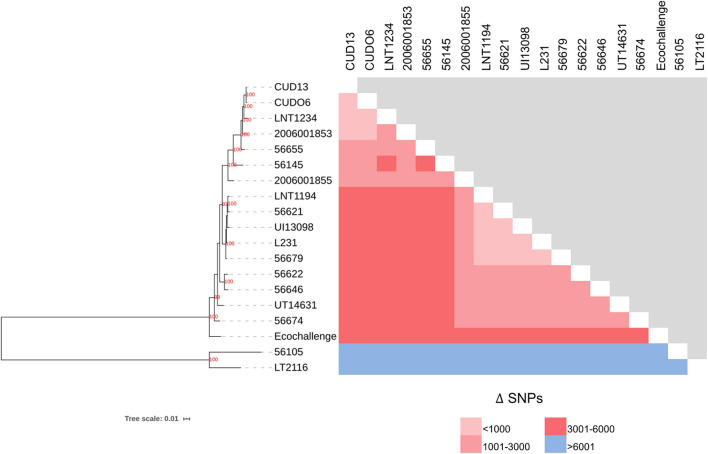
Phylogenetic (ML) analysis, based on concatenated core-SNPs, and the number of different SNPs among 19 *L. weilii* strains. The phylogenetic tree was constructed using the ML method with 1,000 bootstrap replicates. The bootstrap values are presented at the node of the tree, while the number of different SNPs in each pair of strains is shown in pink and blue boxes. The *L. weilii* serogroup Mini strains CUDO6 and CUD13 were phylogenetically clustered into the same clade (100% bootstrap support), demonstrating their close genetic relationship. In addition, they have a small number of different SNPs (39 sites) between them compared to the other strains.

The 19 *L. weilii* genomes analyzed contained a total of 40,599 SNPs in their core-genome region (core-SNPs), with the number of different SNPs between paired genomes varying from 39 to 31,428 SNP sites (Supplementary Table 11). Notably, *L. weilii* strains 56105 and 56655, which were isolated from human leptospirosis in Indonesia and China, respectively, harbored the largest number of different SNPs sites in their core-genome (Figure 5 and Supplementary Table 11). This finding may imply the specific character of these two strains that presumably was related to the number of accumulated SNPs, leading to significant evolutionary diversification from the others. The strains CUDO6 and CUD13 had 39 different SNP sites between them. Moreover, the ML-based tree grouped these strains in the same clade with strong bootstrap support (100%), supporting their close genetic relationship. They clustered in the same lineage as the human *L. weilii* strains from Thailand (strain 2006001853), Laos (strain LNT1234), and China (strain 56655) (Figure 5). Thus, the progenitor of this lineage may have circulated amongst these localities. However, strains CUDO6 and CUD13 showed a distinct genetic distance, in terms of the SNP distance and phylogeny, from the human *L. weilii* strains, which may reflect the evolutionary diversification and adaptation of both strains to infect canine hosts.

### Pan-Genome Analysis and Unique Gene Identification

**FIGURE 6:**
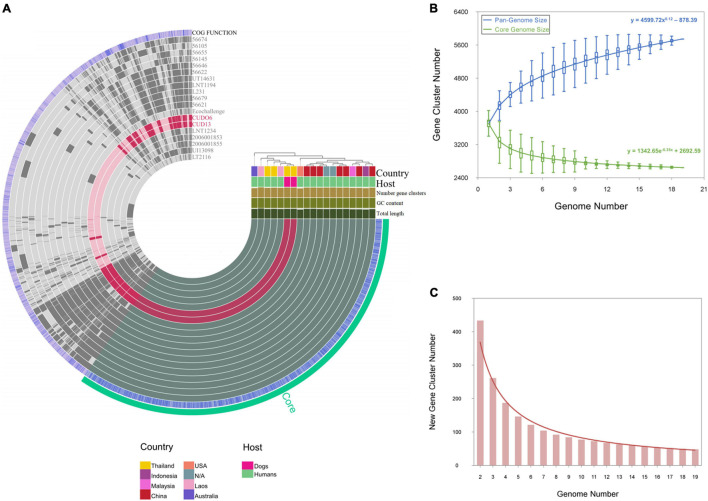
Pan-genome and core-genome analysis of 19 *L. weilii* strains. The genomes in this figure are ordered according to their phylogenomic analysis based on clustering of the presence and absence gene matrix, which is shown at the right upper of figure. The bars in the 19 first layers represent the gene clusters, as calculated by the Euclidian distances across 19 *L. weilii* genomes. The next blue layer demonstrates the functional annotation of gene clusters using the EggNOG database, and the last green layer corresponds to core-genome organization **(A)**. The power-fit and exponential fitting curves with equations of the pan- and core-genomes of the 19 *L. weilii* are represented by blue and green lines, respectively **(B)**, while the new gene curve is manifested by a brown line **(C)**.

Pan-genome analysis revealed that the gene repertoire of the 19 *L. weilii* genomes analyzed was comprised of a total of 5,744 orthologous gene clusters. Of these, 2,645 (46.04%) and 2,183 (38%) gene clusters were identified as belonging to the core and accessory genomes, respectively. The number of strain-specific gene clusters ranged from two in strain CUD13 to 269 in strain LT2116 (Figure 6A). The power-law fitting curve for the pan-genome of *L. weilii* appeared to be expanded after the continual addition of new genomes. In contrast, the trajectory of the exponential fit curve for the core-genome became constant after 17 genomes were analyzed (Figure 6B).

According to the Heaps’ law, the pan-genome of the 19 *L. weilii* analyzed showed characteristics of an “open” pan-genome structure as supported by the γ parameter (γ = 0.12). Consistently, the new gene curve does not reach zero and contributed an average of 48 new genes in each sequential genome addition (Figure 6C). These results are consistent with the previous findings that described the open-pan genome nature of *Leptospira* (Fouts et al., 2016; Xu et al., 2016; Vincent et al., 2019), and correlated with their lifestyle and ability to successfully colonize and infect a broad range of hosts and environments.

A total of 1,946 orthologous gene clusters (73.57%) of the core-genome of *L. weilii* were functionally annotated based on the COG category classification (Supplementary Table 12). Of these, the core-genome of the 19 *L. weilii* analyzed was abundant in encoded proteins related to categories R (general function prediction only, 9.18%), T (signal transduction, 8.39%), M (cell wall/membrane/envelop biosynthesis, 8.05%), and J (translation, 6.42%). This result may explain the common abilities of *L. weilii* that permit this microorganism to sense, respond, replicate, and survive in various hosts and environmental conditions.

Furthermore, the pan-genome analysis revealed strain-specific genes that were uniquely presented in strains CUDO6 and CUD13. Strain CUDO6 carried two strain-specific genes with potential hits in the VFDB of the twitching motility protein (30% amino acid identity) and auto-transport protein (29% amino acid identity) of *Burkholderia pseudomallei* K96243. Strain CUD13 contained three strain-specific genes, one as a hypothetical protein of unknown function, and the other two genes resembling the ABC transporters of *Klebsiella pneumoniae* NTUH-K2044 (28 and 31% amino acid identity, respectively).

### Variant Calling Analysis

In comparison with *L. weilii* strain 2006001853 isolated from human leptospirosis in Thailand, a total of 1,101 missense mutations were identified amongst both *L. weilii* CDSs. Of these, 591 CDSs were functionally categorized in the COG database and those related to categories S (function unknown), M (cell wall/membrane/envelope biogenesis), T (signal transduction mechanisms), and E (amino acid transport and metabolism) were over-assigned in the set of missense variants, accounting for the majority of protein function that was affected (Supplementary Table 13). Interestingly, there were instances of missense mutations in pathogenesis-related genes involved with the endoflagellum (*flaA2*, *flaB2*, *fliD*, *fliE*, *fliO*, *flgD*, *flhA*, and *flhB*), LPS structure (*degT*, *fcl*, *neuB1, neuB2, rfaD*, *rfbB, lptD, lpxD1*, *lpxD2, wzx*, and *wzy*), mammalian cell entry protein (*mce*), and hemolytic and sphingomyelinase activities (*sph1*, *sph2*, and *tlyA*) in strains CUDO6 and CUD13. The missense mutations included SNPs, insertion/deletion (indels), and complex variations (when more than one change occurred in the same gene) (Table 2). In endoflagellum and LPS structure, all missense mutation types were found amongst pathogenesis-related genes. The gene encoding for flagellar type III secretion system (*flhB*) showed mutations consisting of four SNPs and two indels, resulting in six amino acid changes. Similarly, *wzx* encoding for O-antigen flippase also showed several mutations consisting of four SNPs, generating four amino acid substitutions. For the mammalian cell entry gene *mce*, a A656G mutation was observed, leading to substitution of lysine to arginine at amino acid position 219. The most complex variations were found in *sph2*, which harbored eight SNPs and five indels, resulting in 19 amino acid changes (Table 2). The missense mutation of genes encoding for virulence determinants of pathogenic *Leptospira* may be one reason for attenuated virulence and asymptomatic infection in animals.

**TABLE 2:** Genetic variation in 23 representative pathogenesis-related genes in *L. weilii* strains CUDO6 and CUD13 comparing to *L. weilii* strain 2006001853 as a reference.

Gene	Gene annotation	Variation^a^		Type of missense variation^b^
		Nucleotide	Amino acid	

Motility				
*flaA2*	Flagellar filament outer layer protein	A86C	Gln29Pro	SNPs
*flaB2*	Flagella filament protein	G34T, **del**AGTA165-168**ins**GATC	Ala12Ser, Val56Ile	Complex
*fliD*	Flagellar filament capping protein	A574G, G1471A	Asn192Asp, Gly491Ser	SNPs
*fliE*	Flagellar hook-basal body complex protein	T313G	Ser105Ala	SNPs
*fliO*	Flagellar biosynthesis protein	T86C	Val29Ala	SNPs
*flgD*	Flagellar hook capping protein	C566T, A707G	Pro189Leu, Gln236Arg	SNPs
*flhA*	Flagellar biosynthesis protein	**del**CGGAG651-655**ins**AGGTT	Ala219ser	Indels
*flhB*	Flagellar type III secretion system	A391C, G653A, G661A, **del**GCT751-753**ins**ACG, C1003T, **del**TG1014-1015**ins**CA	Asn131His, Ser218Asn, Ala221Thr, Ala251Thr, Leu335Phe, Val339Ile	Complex

**LPS structure**				

*degT*	DegT aminotransferase	A158G, A368G	Asp53Gly, Asp123Gly	SNPs
*fcl*	dTDP-4-dehydrorhamnose reductase	C589T, G893A	Leu197Phe, Gly298Glu	SNPs
*neuB1*	*N*-acetyl neuraminic acid synthetase 1	G132C, C443T, G685A	Glu44Asp, Ala148Val, Ala229Thr	SNPs
*neuB2*	*N*-acetyl neuraminic acid synthetase 2	A643G, **del**GATG654-657**ins**AATC, A731G	Ile215Val, Met219Ile, Glu244Gly	Complex
*rfaD*	ADP-glyceromanno-heptose 6-epimerase	T899C	Phe300Ser	SNPs
*rfbB*	dTDP-glucose 4,6-dehydratase	G600A	Met200Ile	SNPs
*lptD*	LPS-assembly protein	G334A	Ala112Thr	SNPs
*lpxD1*	UDP-3-*O*-(3-hydroxymyristoryl) glucosamine *N*-acyltransferase	**del**AGAG1014-1017**ins**GGTT	Glu339Val	Indels
*lpxD2*	UDP-3-*O*-(3-hydroxymyristoryl) glucosamine *N*-acyltransferase	C394A	Gln132Lys	SNPs
*wzx*	O-antigen flippase	A1210G, A1284C, T1297C, T1306C	Ile404Val, Leu428Phe, Phe433Leu, Phe436Leu	SNPs
*wzy*	O-antigen polymerase	G779A, T1201G	Arg260Gln, Ser401Ala	SNPs

**Mammalian cell entry**				

*mce*	Mammalian cell entry protein	A656G	Lys219Arg	SNPs

**Exotoxin**				

*sph1*	Sphingomyelinase C 1	G1186A	Ala396Thr	SNPs
*sph2*	Sphingomyelinase C 2	C692T, **del**AAC708-710**ins**GAT, A727G, **del**CCATTCAT735-742**ins**TAGTCCAG, A789G, A808G, **del**CCATTCAT816-823**ins**TAGTCCAG, **del**GACCG836-840**ins**AATCA, G868C, C1151T, C1979T, **del**GG2032-2033**ins**AA, A2152G	Thr231Ile, IleThr236MetIle, Ile243Val, HisSerSer246SerProAla, Ile263Met, Ile270Val, HisSerSer273SerProAla, ArgPro279GlnSer, Glu290Gln, Ala384Val, Ala660Val, Gly678Asn, Ile718Val	Complex
*tlyA*	Hemolysin A	C227T, A464G	Ala76Val, His155Arg	SNPs

*^*a*^**del**, nucleotide deletion; **ins**, nucleotide insertion.*

*^*b*^SNPs, single nucleotide polymorphisms; indels, insertion/deletion of nucleotides.*

Endoflagella-mediated motility is a crucial VF for pathogenic *Leptospira* (Gomes-Solecki et al., 2017). Constructed mutant strains with affected endoflagella-related genes (*flaA2* and *fcpA*) showed a loss of translocation and ability to infiltrate the host cell (Lambert et al., 2012; Wunder et al., 2016). Moreover, a spontaneous non-motile mutant strain isolated from a dog with leptospirosis contained a single nucleotide deletion in *fliM*, resulting in the absence of motility in soft agar and lack of pathogenicity to cause disease in hamsters (Fontana et al., 2016). To examine the effect of the missense mutations presented in endoflagella genes on motility, motility of *L. weilii* strains CUDO6 and CUD13 was tested and compared with the motile reference *L. interrogans* serovar Copenhageni strain M20. Motility was observed for the *L. interrogans* control strain, which swarmed around 1 cm away from the inoculation spot, but not for *L. weilii* strains CUDO6 and CUD13 after 5 days of incubation (Supplementary Figure 6). Additionally, *L. weilii* strains CUDO6 and CUD13 reached a similar OD of 0.4–0.5 as *L. interrogans* serovar Copenhageni strain M20 after 5 days of incubation, indicating that the observed absence of motility was not due to a slower growth rate of the *L. weilii* strains. Although none of the missense mutations resulted in endoflagellar gene truncation, the result provides a plausible explanation that the missense mutations observed in the endoflagella genes of *L. weilii* strain CUDO6 and CUD13 might impair movement function, decrease systemic bacterial dissemination, and facilitate host adaptation during asymptomatic infection. Similarly, LPS has been described as an essential factor for *Leptospira* infection (Gomes-Solecki et al., 2017). This complex cell wall component comprises three parts (lipid A moiety, core oligosaccharide, and O-antigen) responsible for antigenic variation and serovar diversity in pathogenic *Leptospira* (Patra et al., 2015). Previously, a *lpxD1-*mutant strain with a hydrophobic modification of lipid A in the LPS structure, showed attenuation of its virulence and gain of temperature adaptation (Eshghi et al., 2015). Additionally, evidence of over-expression of the O-antigen was associated with chronic infections in a rat model (Nally et al., 2005). Both findings could imply that alteration of the genes in the LPS structure may affect LPS expression and allow *Leptospira* to persist in animal carriers.

The *mce* gene encodes for mammalian cell entry protein and has a role in cell adherence and invasiveness in *Leptospira* (Zhang et al., 2012). Accordingly, a *mce*-mutant strain was demonstrated to have an attenuated phenotype with a decreased ability to internalize into macrophages during the early stage of infection (Zhang et al., 2012). Moreover, a *mce*-mutant strain was detected in the urine of an animal model up until 2 weeks of post-infection (Zhang et al., 2012). This figure reflects the possibility that genetic mutation of *mce* may allow the bacterium to survive during infection and provide advantage associated asymptomatic carriage in animals. Pathogenic *Leptospira* can produce hemolysins that target red blood cells and host cell membrane sphingomyelin for nutrient acquisition and induce pro-inflammatory cytokines during the early stage of infection (Wang et al., 2012). However, the *sph2*-mutant strain had almost no hemolytic and sphingomyelinase activities, suggesting that genetic mutation of *sph2* may reduce the function of hemolysins in pathogenic *Leptospira* (Narayanavari et al., 2015).

During chronic infection, pathogenic *Leptospira* will compete with selective pressures, arising from host immune response and changes in different ecological niches. Several adaptive strategies are used for short- and long-term adaptations, including altered VFs and/or the formation of factors that enhance their capacity to persist in the host. In short-term adaptation, many regulatory mechanisms are believed to quickly adjust gene expression to down-regulate their virulence and/or increase expression of genes related to host persistence, such as O-antigen and biofilm (Nally et al., 2005; Yamaguchi et al., 2018). Altogether, genetic alterations that affect protein function (i.e., missense mutation) may be linked to long-term adaptation, especially to beneficial mutations that are the consequence of positive or diversifying selection (Xu et al., 2016). This advantageous selection may either enforce augmentation of persistence factors and/or repression of VFs during asymptomatic infection, leading to an inheritable shift in the bacterial population to a less pathogenic state in the host (Kurilung et al., 2019). However, the association of genetic mutations with leptospiral asymptomatic infection in dogs remains to be elucidated in further studies.

## Conclusion

This study provided new serotyping and genomic analyses insight into the two *L. weilii* strains (CUDO6 and CUD13) that were isolated from the urine of asymptomatic dogs. Both strains were of the same serogroup (serogroup Mini), with almost conserved genomic features. They had a close evolutionary relationship based on their core-SNPs phylogeny, reflecting the same microevolutionary background. Nevertheless, some disparities were identified amongst their genomes, including the chromosomal rearrangement, plasmid sequence, and unique genes encoding for twitching motility protein, auto-transport protein, and ABC transporter protein. Compared with the *L. weilii* strain from human, missense mutations in pathogenesis-related genes encompassing endoflagella, LPS structure, mammalian cell entry, and hemolytic activities were identified in non-motile CUDO6 and CUD13. These may be involved with the host-adaption of the strains to enable them to persist as an asymptomatic infection in dogs. Overall, this study provides important genetic information to explore further the molecular epidemiology and the host-pathogen interactions of pathogenic *L. weilii* species.

## Data Availability Statement

The datasets presented in this study can be found in online repositories. The names of the repository/repositories and accession number(s) can be found in the article/Supplementary Material.

## Author Contributions

AK conceived and designed the experiments, performed the experiments, analyzed the data, and contributed to the writing of the manuscript. VP and NP contributed to study design, reagents, materials, and analysis tools, and supervision and proofreading of the writing. All authors contributed to the article and approved the submitted version.

## Conflict of Interest

The authors declare that the research was conducted in the absence of any commercial or financial relationships that could be construed as a potential conflict of interest.

## Publisher’s Note

All claims expressed in this article are solely those of the authors and do not necessarily represent those of their affiliated organizations, or those of the publisher, the editors and the reviewers. Any product that may be evaluated in this article, or claim that may be made by its manufacturer, is not guaranteed or endorsed by the publisher.

## Footnotes

1
https://github.com/rrwick/Porechop

2
https://www.bioinformatics.babraham.ac.uk/projects/fastqc/

3
https://github.com/tseemann/snippy

4
http://tree.bio.ed.ac.uk/software/figtree/

5
https://github.com/ekg/freebayes
