# Supplementary material for: Comparative Genomic Analysis and a Novel Set of Missense Mutation of the Leptospira weilii Serogroup Mini From the Urine of Asymptomatic Dogs in Thailand
Source: Front Microbiol. 2021 Oct 18;12:731937. doi: 10.3389/fmicb.2021.731937 (PMC8558515; doi:10.3389/fmicb.2021.731937)
Supplement: Supplementary file 2 [file Data_Sheet_2.docx]

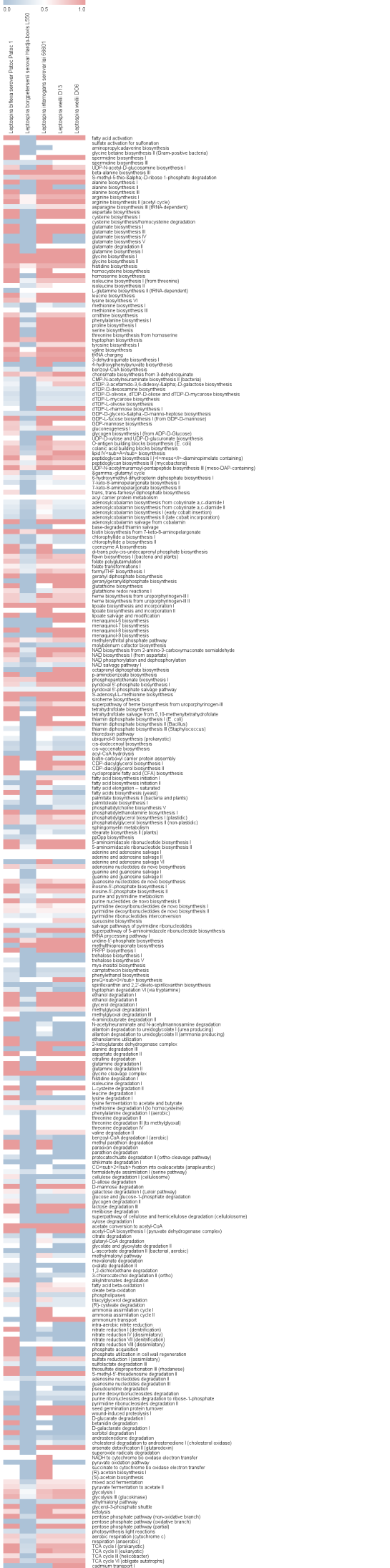
**Supplementary data 2**

**Comparative genomic analysis and a novel set of missense mutation of the *Leptospira weilii* serogroup Mini from the urine of asymptomatic dogs in Thailand**

**Alongkorn Kurilung^a,b,c^, Vincent Perreten^b,*^ and Nuvee Prapasarakul^a,d,*^**

*^a^Department of Veterinary Microbiology, Faculty of Veterinary Science, Chululongkorn University, Bangkok 10330, Thailand*

*^b^Institute of Veterinary Bacteriology, Vetsuisse Faculty, University of Bern, CH-3001 Bern, Switzerland*

*^c^Siriraj Metabolomics and Phenomics Center, Faculty of Medicine Siriraj Hospital Mahidol University, Bangkok 10700, Thailand*

*^d^Diagnosis and Monitoring of Animal Pathogen Research Unit, Faculty of Veterinary Science, Chulalongkorn University, Bangkok 10330, Thailand*

**Corresponding authors.*

*Vincent Perreten*

*E-mail address: vincent.perreten@vetsuisse.unibe.ch.*

*Nuvee Prapasarakul*

*E-mail address: Nuvee.P@chula.ac.th.*


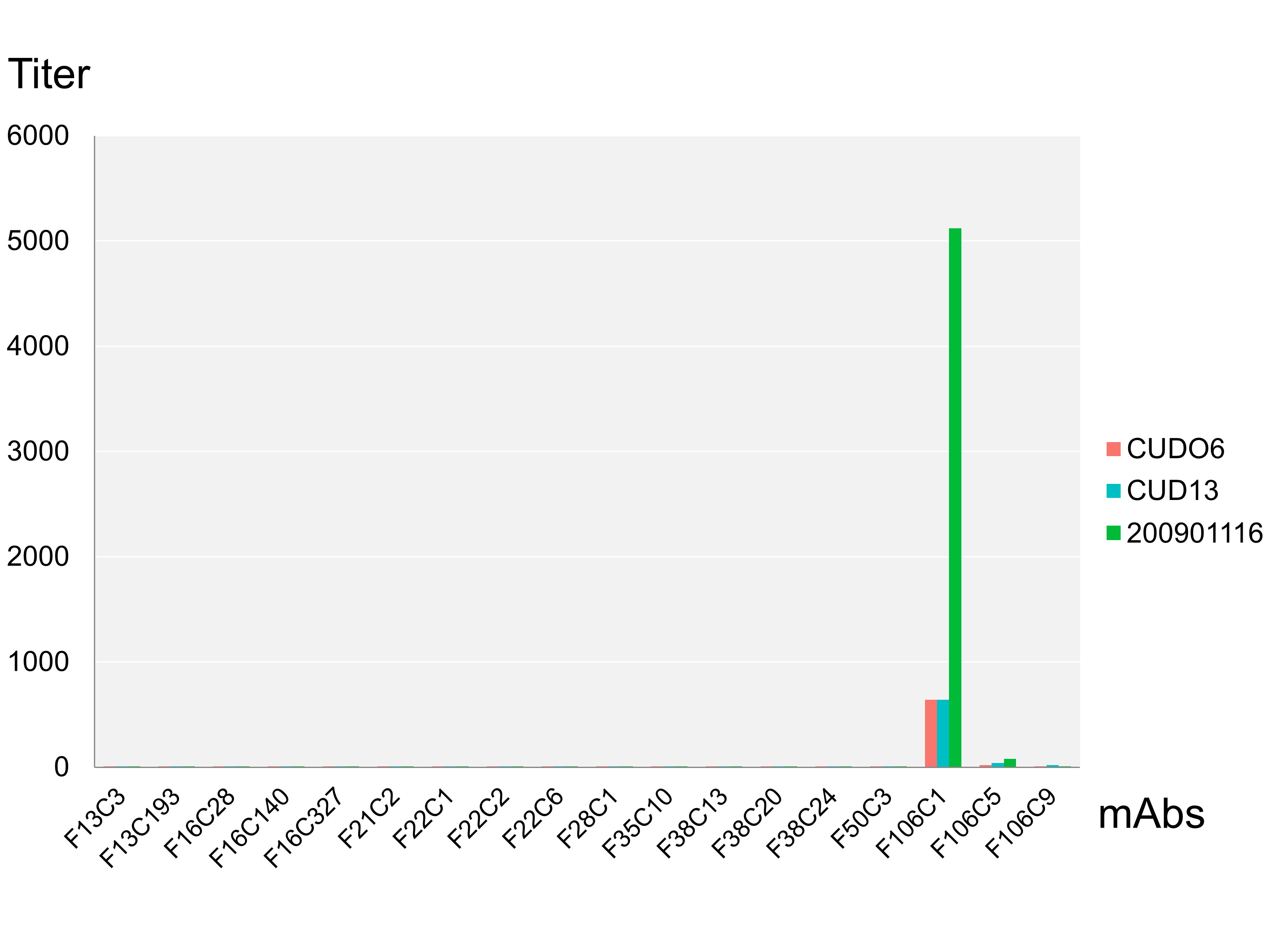


**Supplementary data 2, Figure S1.** Serological classification of undesignated serovar of *Leptospira* serogroup Mini using monoclonal antibodies typing. *L. weilii* strains CUDO6 (pink bar) and CUD13 (blue bar) had the highest MAT titer for monoclonal antibody F106C1 than that those others (1:640). They had closely antigenic relatedness with undesignated serovar of *L. mayottensis* strain 200901116 as indicated by closely agglutination pattern of monoclonal antibodies typing.


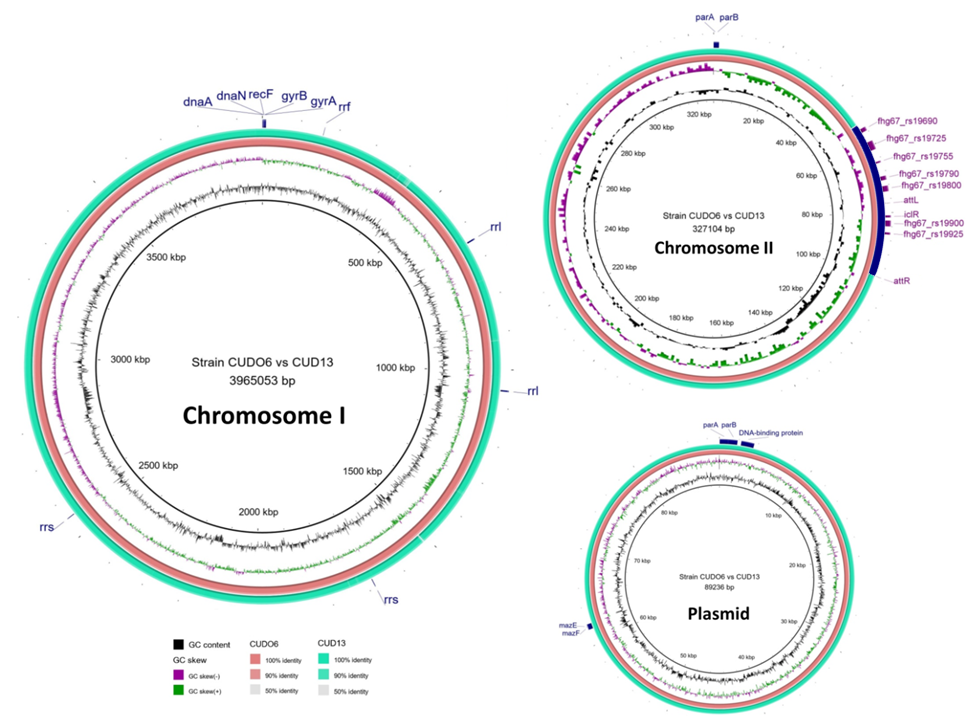


**Supplementary data 2, Figure S2.** Circular genome comparison of *Leptospira weilii* strains CUDO6 and CUD13. The comparative nucleotide sequence analysis was performed with BRIG (Alikhan et al., 2011). The second and third inner rings indicate GC content and GC skew, respectively. The fourth and fifth colored rings represent chromosomal DNA (chromosome I and chromosome II) and plasmid of the strain CUDO6 (Geraldine) and CUD13 (shamrock green). The outermost labels reveal location of genes involved with *ori* and partition systems (*dnaA*, *dnaN*, *recF*, *gyrA*, *gyrB*, *parA*, and *parB*), ribosomal genes (*rrf*, *rrl*, and *rrs*), prophage-associated genomic island (*fhg67_rs19690*, *fhg67_rs19725*, *fhg67_rs19755*, *fhg67_rs19790*, *fhg67_rs19800*, *fhg67_rs19900*, *fhg67_rs19925*, *attL*, *attR*, and *iclR*), and Toxin-antitoxin system (*mazF and mazE*).

**
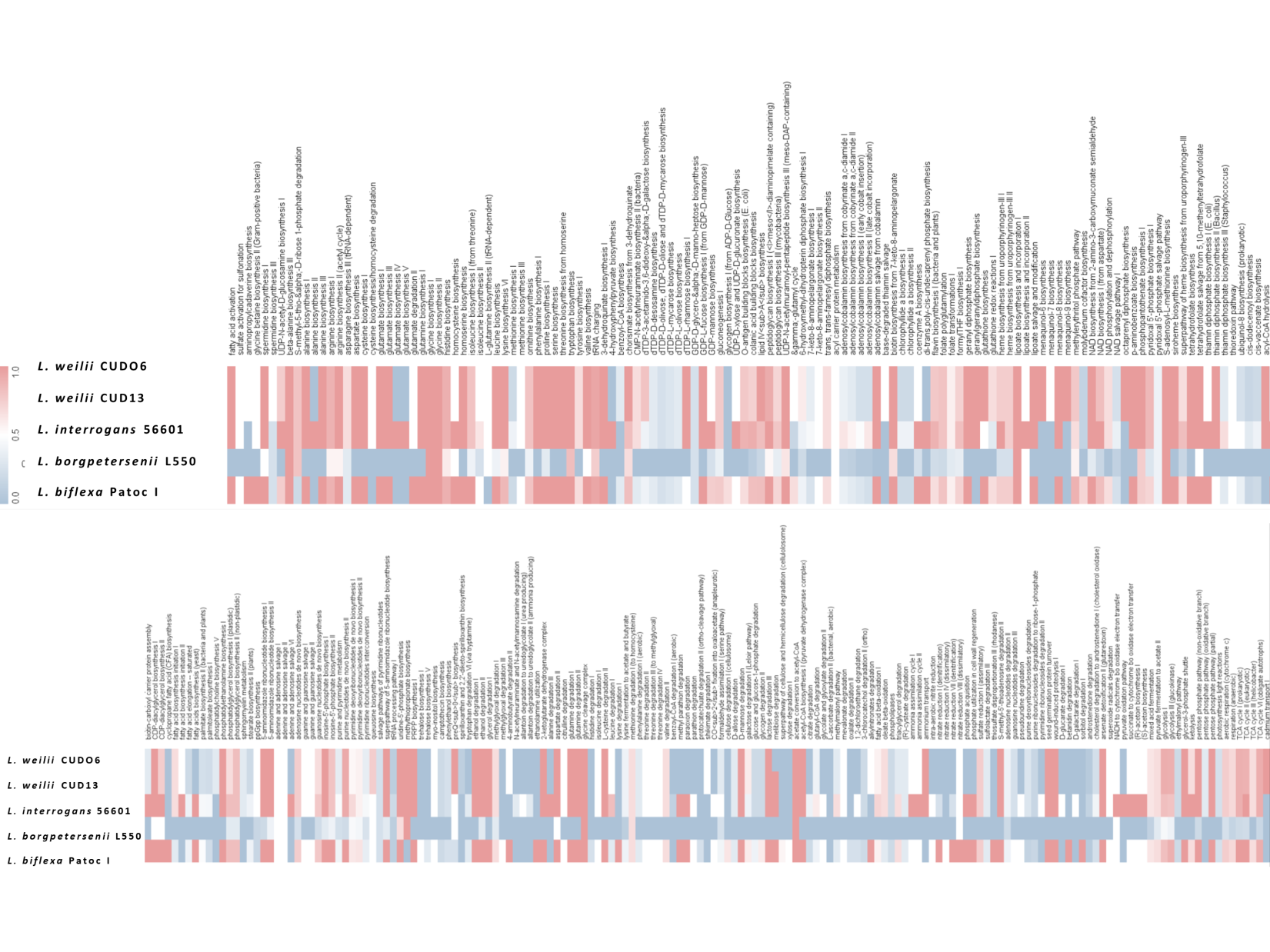
Supplementary data 2, Figure S3.** Comparison of KEGG metabolic profiles in *Leptospira weilii* strains CUDO6 and CUD13 and other *Leptospira* species (*n*=3). The intensity of pink and blue color represents pathway completion value from 0 to 1. The zero value indicates absence of genes in a given pathway, and the one value indicates complete of gene set in the pathway.


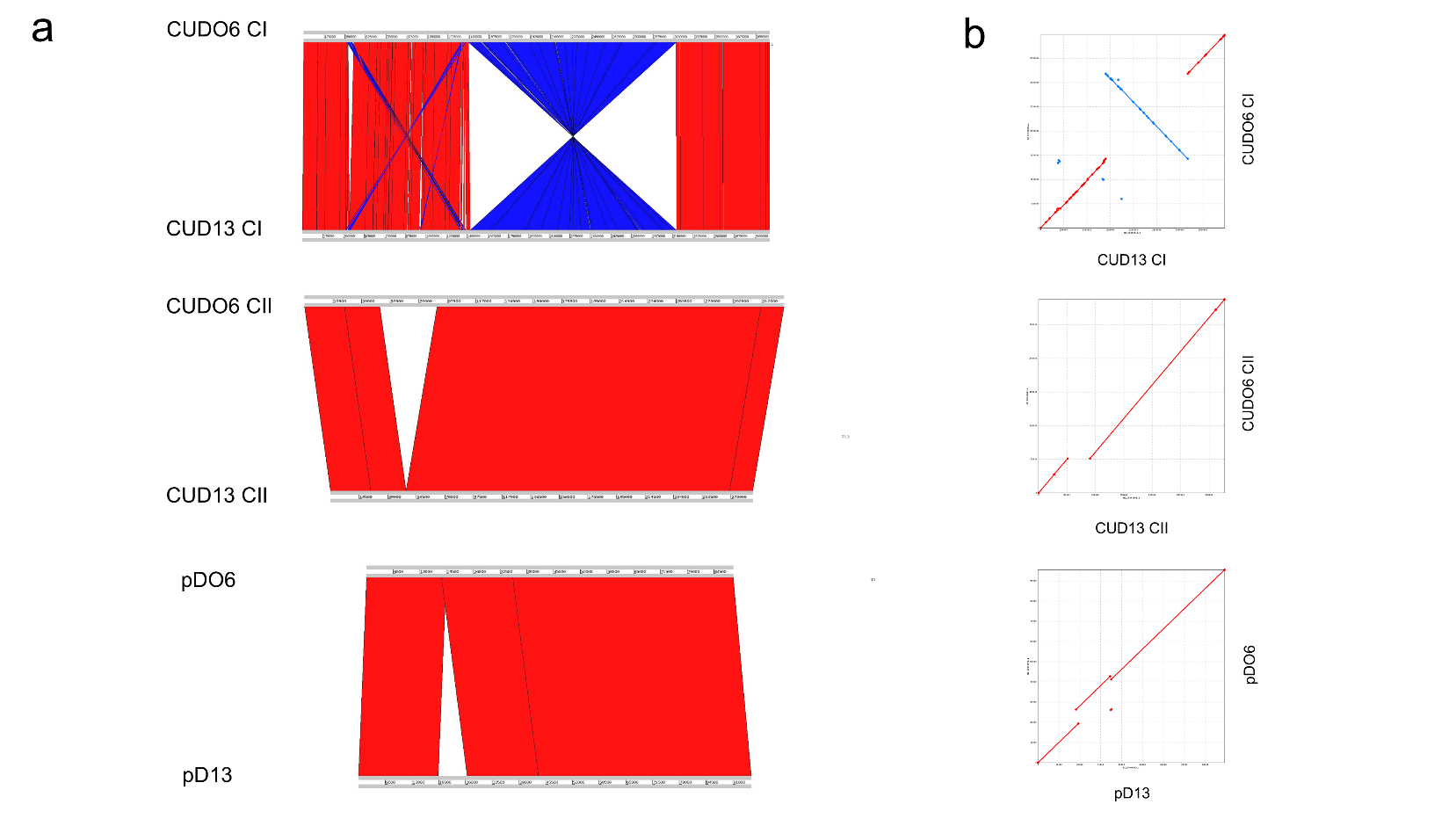


**Supplementary data 2, Figure S4. Genome comparisons and dot plots analysis of *L. weilii* strains CUDO6 and CUD13.** Sequences were aligned from the predicted replication origin of each replicons and visualized using ACT with a cut-off of BLAST scores >500 (Carver et al., 2005). Red bands represent similar regions with the same orientation and blue bands represent regions with inversion (a). Dot plots comparison were generated using MUMmer4 with the default parameter setting (Marcais et al., 2018). The red dots represent matched regions between comparing replicons and blue dots represent match region with inversion (b).


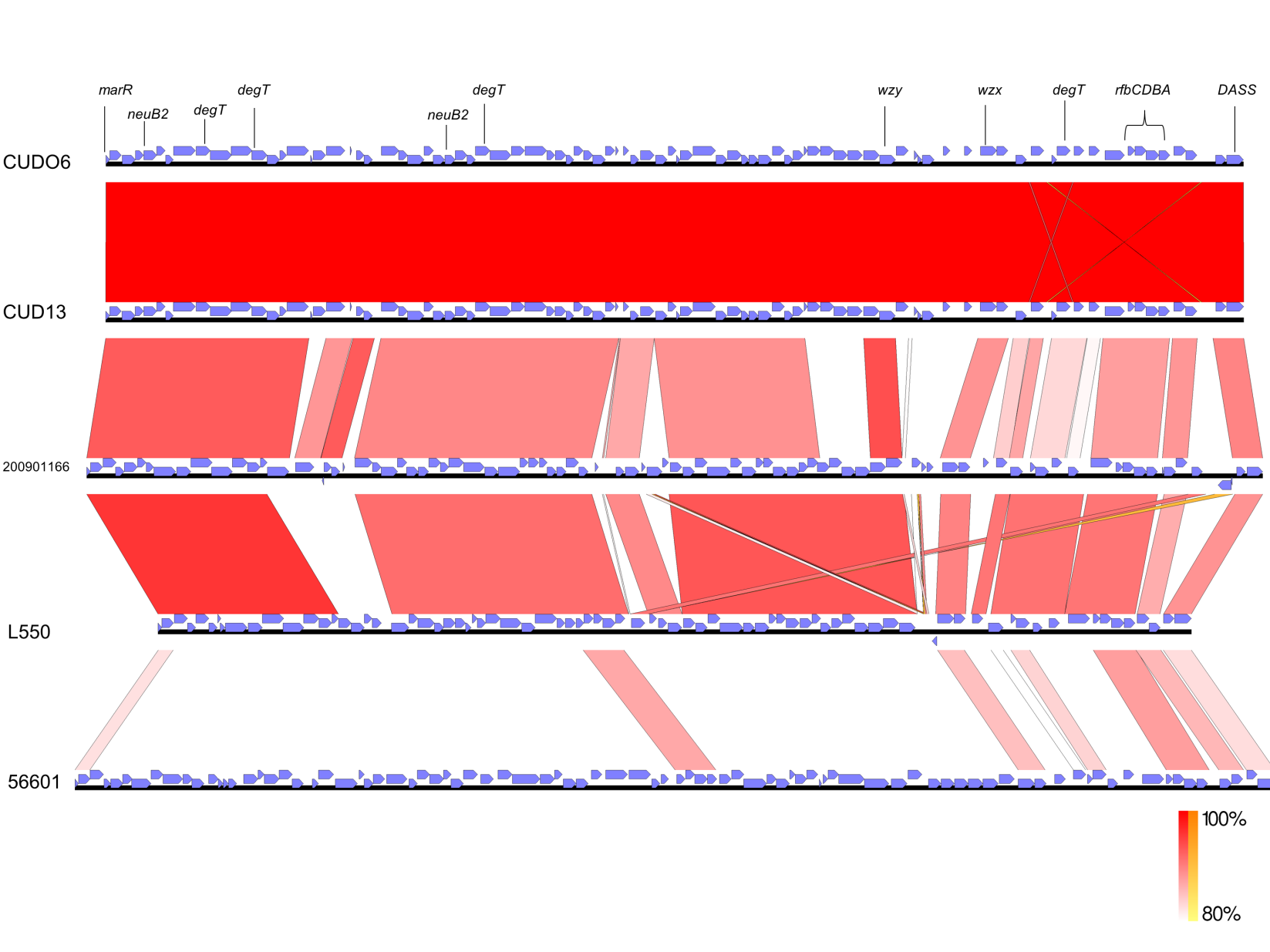


**Supplementary data 2, Figure S5.** Comparison of serovar determinant region (*rfb* locus) in *Leptospira* serogroups Mini (CUDO6, CUD13 and 20090166), serogroup Hardjo (L550) and serogroup Icterohaemorrhagiae (56601). The fiure was generated using Easyfig program (Sullivan et al., 2011).The *rfb* region share genes encoding for transcriptional regulaor (*marR*) and sodium anion symportor (*DASS*) in the upstream and downstream of the locus, respectively. Moreover, rhamnose biosynthesis gene cluster (*rfbABCD*) are conseved between difference serogroups. *L. weilii* serogroup Mini strains CUDO6 and CUD13 show closely relationship of *rfb* locus with *L. mayotensis* serogroup Mini strain 20090166.


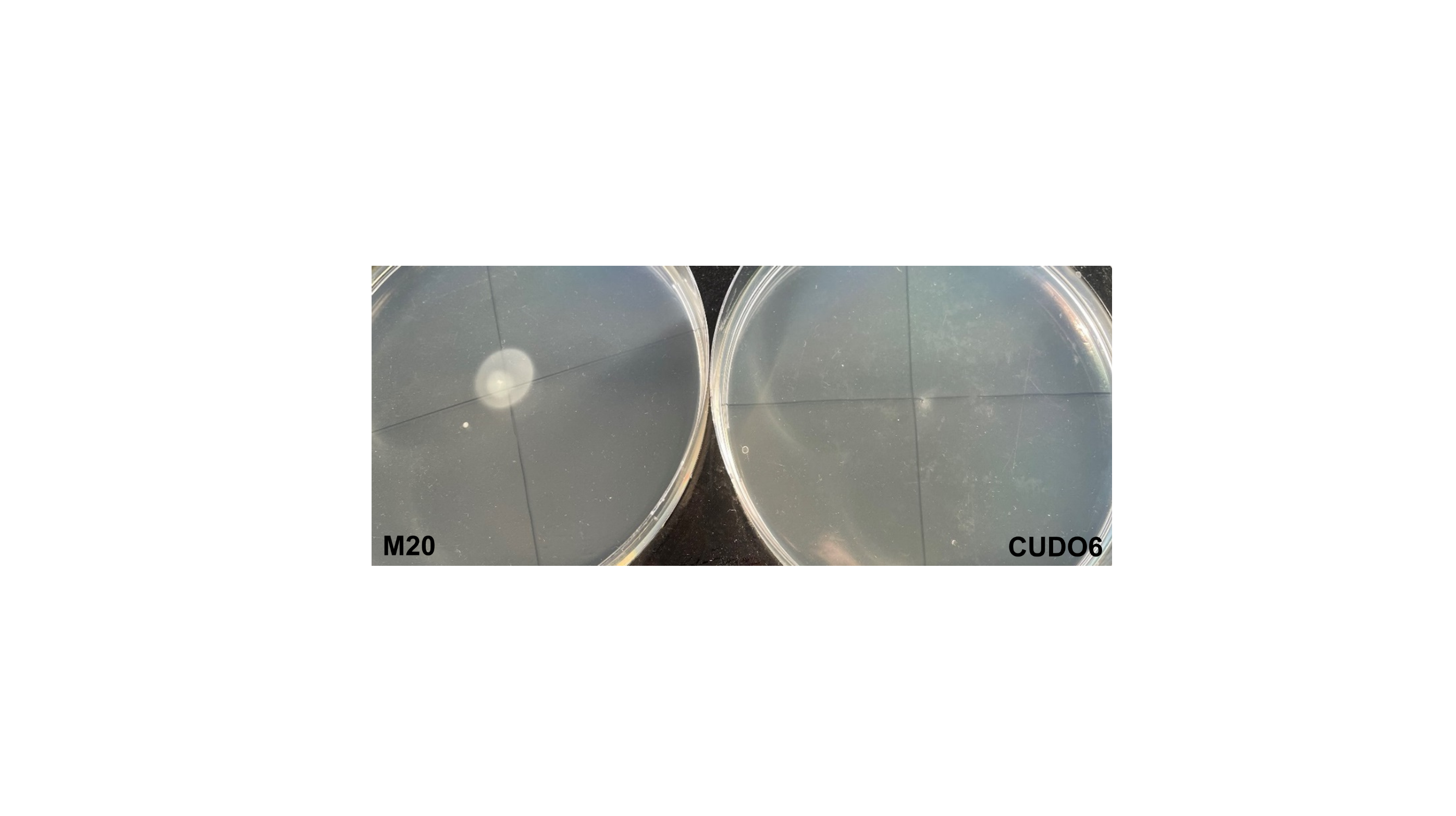


**Supplementary data 2, Figure S6. Motility assay of the *L. weilii* strains CUDO6, and** ***L. interrogans* serovar Copenhageni strain M20 motile control strain.** *Leptospira* was spotted onto soft agar (0.5%) EMJH, and incubated at 30°C for five days. The ability to swarm on soft agar was observed from the inoculation point. *L. weilii* strains CUDO6 showed no migration from the inoculation site, while *L. interrogans* serovar Copenhageni strain M20 exhibited strongly swarming of around 1 cm away from the inoculation spot. The same was observed for *L. weilii* strains CUD13.

**References**

Alikhan, N.F., Petty, N.K., Ben Zakour, N.L., and Beatson, S.A. (2011). BLAST Ring Image Generator (BRIG): simple prokaryote genome comparisons. *BMC Genomics* 12**,** 402.

Carver, T.J., Rutherford, K.M., Berriman, M., Rajandream, M.-A., Barrell, B.G., and Parkhill, J. (2005). ACT: the Artemis comparison tool. *Bioinformatics* 21**,** 3422-3423.

Marcais, G., Delcher, A.L., Phillippy, A.M., Coston, R., Salzberg, S.L., and Zimin, A. (2018). MUMmer4: A fast and versatile genome alignment system. *PLoS Comput Biol* 14**,** e1005944.

Sullivan, M.J., Petty, N.K., and Beatson, S.A. (2011). Easyfig: a genome comparison visualizer. *Bioinformatics* 27**,** 1009-1010.
